# Chemistry of Protein-Phenolic Interactions Toward the Microbiota and Microbial Infections

**DOI:** 10.3389/fnut.2022.914118

**Published:** 2022-07-01

**Authors:** Hilal Yilmaz, Busra Gultekin Subasi, Hasan Ufuk Celebioglu, Tugba Ozdal, Esra Capanoglu

**Affiliations:** ^1^Department of Biotechnology, Faculty of Science, Bartin University, Bartin, Turkey; ^2^Division of Food and Nutrition Science, Chalmers University of Technology, Gothenburg, Sweden; ^3^Hafik Kamer Ornek MYO, Sivas Cumhuriyet University, Sivas, Turkey; ^4^Department of Food Engineering, Faculty of Engineering and Natural Sciences, Istanbul Okan University, Istanbul, Turkey; ^5^Department of Food Engineering, Faculty of Chemical and Metallurgical Engineering, Istanbul Technical University, Istanbul, Turkey

**Keywords:** food phenolics, protein-phenolic interaction, antiviral, antioxidant activity, phytochemicals, microbial protein (MP)

## Abstract

Along with health concerns, interest in plants as food and bioactive phytochemical sources has been increased in the last few decades. Phytochemicals as secondary plant metabolites have been the subject of many studies in different fields. Breakthrough for research interest on this topic is re-juvenilized with rising relevance in this global pandemics' era. The recent COVID-19 pandemic attracted the attention of people to viral infections and molecular mechanisms behind these infections. Thus, the core of the present review is the interaction of plant phytochemicals with proteins as these interactions can affect the functions of co-existing proteins, especially focusing on microbial proteins. To the best of our knowledge, there is no work covering the protein-phenolic interactions based on their effects on microbiota and microbial infections. The present review collects and defines the recent data, representing the interactions of phenolic compounds -primarily flavonoids and phenolic acids- with various proteins and explores how these molecular-level interactions account for the human health directly and/or indirectly, such as increased antioxidant properties and antimicrobial capabilities. Furthermore, it provides an insight about the further biological activities of interacted protein-phenolic structure from an antiviral activity perspective. The research on the protein-phenolic interaction mechanisms is of great value for guiding how to take advantage of synergistic effects of proteins and polyphenolics for future medical and nutritive approaches and related technologies.

## Introduction

Phenolic compounds are secondary metabolites that are synthesized through the shikimic acid and phenylpropanoid pathways found in most plant tissues, including fruits and vegetables. Even though they are not one of the major nutrients, they provide many bioactive properties including antioxidant, anti-cancer, anti-inflammatory, antimicrobial properties. Their bioactive properties provide many health-protective effects including prevention of cancer, cardiovascular diseases, diabetes, and stroke (1).

Protein-phenolic interactions take place in two different mechanisms, which include either covalent or non-covalent interactions. Covalent bonding between two molecules of proteins and phytochemicals is accomplished by two mechanisms, enzymatic and non-enzymatic reaction mechanisms; on the other hand, non-covalent interactions include hydrogen bonding, hydrophobic interactions, electrostatic interactions, and van der Waals binding forces that are reversible reactions. Additionally, some internal factors including characteristics of proteins, types of phytochemicals, protein/phytochemical ratio and external factors including temperature, pH, ionic strength, additional reagents, and other food components influence these interactions (2).

The reciprocal interactions between phenolic compounds and proteins result in various nutritional, functional, and structural changes in both sides. Among these properties, antioxidant activities are of significant importance in terms of organic chemistry, as well as health and nutrition. Mainly, all antioxidative compounds take the action throughout some particular mechanisms which are scavenging of the free radicals in the media, inhibition of enzymes causing free radical formation, chelating the existing metallic ions, induction of endogenous antioxidant enzymes, preventing the lipid peroxidation, DNA damage, protein modification, and/or sugar degradation (3). All these mechanisms are attributed with many diseases within the living organisms; hence, dietary antioxidants have crucial importance to be a part of the diet (4). Dietary phenolic compounds, due to their antioxidant potentials besides their other benefits have been investigated extensively. In one-step further, reaction of dietary phenolic compounds with protein structures might increase the antioxidative properties of phenolics (5). Particularly, flavonoids and phenolic acids have been subjected to extensive studies in order to clarify the reaction mechanisms with protein structures and the consequences of these interactions in terms of biological activities.

Oxidative stress induced by viruses is well established. The viral infection interferes with the body's important metabolic processes in addition to participating in the replication of the virus (6). In this sense, not only the antioxidant properties but also the antiviral activities of phenolic compounds have great significance especially during the recent pandemics of viral diseases. Phenolic compounds have potential to inhibit viral replication by regulating viral adsorption, binding to cell receptors, inhibition of virus penetration into the host cell and by competing for pathways of activation of intracellular signals (7). However, the existing studies have focused on the antiviral effects of phenolics alone; thus, the antiviral mechanisms of the protein-phenol conjugates are not fully elucidated. Similarly, it is known that dietary phenolic compounds affect both physiology and gene expressions of gut microorganisms. These effects can result from interactions of phenolic compounds with bacterial proteins.

To the best of our knowledge, there is no recent study framing the chemistry of food protein - phenolic interactions playing roles in their antioxidant and antimicrobial activities. Thus, the present review collects and defines the recent data, representing the interactions of phenolic compounds, mainly flavonoids and phenolic acids with food proteins and explores how these molecular-level interactions account for the human health directly and/or indirectly, such as increased antioxidative properties, altering physiology of gut microorganisms and antimicrobial capabilities. As antimicrobial mechanism of food protein-phenol conjugates is not clear with the existing literature, we focused on the microbial protein– dietary phenol interactions in the present review to better understand the chemistry of protein-phenolic binding.

## Chemistry of Protein-Phenolic Interactions

Protein-phenolic interactions take place as covalent and non-covalent interactions (8, 9). Covalent interaction mechanisms are defined as the establishment of irreversible binding between molecules under specific conditions such as availability of phenolic oxidases or alkaline conditions. Non-covalent interactions are defined as the reversible forces including electrostatic interactions, hydrogen bonding, hydrophobic interactions, and ionic bonds (8, 10). These reciprocal actions between proteins and phenolics result in a change in the nutritional, functional, and biological characteristics of both proteins and phenolics.

Covalent bonding between two molecules of proteins and phytochemicals is accomplished by two reaction mechanisms: Enzymatic and non-enzymatic reaction mechanisms. Enzymatic reaction mechanisms mostly use phenolic oxidase such as laccase and tyrosinase. In enzymatic reaction mechanisms, non-enzymatic processes are used to convert phenolics to semiquinone or quinone intermediates. These semiquinone and quinone intermediates are then assailed by nucleophilic side chains and produce C–S or C–N covalent bonds between molecules (10, 11). For non-enzymatic protein-phenolic reaction mechanisms, the free radical grafting and alkaline reaction are two extensively utilized procedures. Phenolic compounds are susceptible to oxidation while in contact with air under alkaline conditions. When they are oxidized, they turn into semiquinones and then to quinones. Covalent cross linkages are formed between proteins-phenolic compounds by reaction of nucleophilic amino acid residues such as Lys, Try, Met and Cys with these extremely reactive products (12, 13). Hydrogen peroxide (H_2_O_2_) and ascorbic acid are frequently used as a redox pair in the free-radical grafting method. They produce hydroxyl radicals, and these radicals oxidize amino acids on the side chain of the proteins. Then, they react with phenolic compounds and generate cross-linked conjugates (14, 15).

Non-covalent interactions include reversible reactions and they contain hydrogen bonding, hydrophobic interactions, electrostatic interactions, and van der Waals binding forces (8, 9). Firstly, phenolic compounds are recognized as hydrogen donors that can produce hydrogen bonds with protein carboxyl groups. Hydrogen bonding occur between hydroxyl groups of phenolics and the oxygen or nitrogen, specifically hydroxyl and amino groups of proteins (10, 16). Specifically, associations between compounds non-polar aromatic ring of the phenolic and hydrophobic regions of the protein molecules are the main reason of hydrophobic interactions (11). Furthermore, electrostatic interactions occur between the hydroxyl groups of phenolics and charged groups on the proteins. In general, a non-covalent protein-phenolic complex is the consequence of a combination of some foregoing relationships, with hydrogen bonding and hydrophobic interactions serving as the primary driving forces. According to the literature, most of the interactions between protein and phenolic compounds occur as non-covalent interactions in nature. Despite its instability, the non-covalent protein-phenolic interaction has a significant influence on the establishment and enhancement of associated food systems in the food industry (17).

In comparison with the non-covalent interactions, covalent interactions are irreversible and more stable in food processes. However, both covalent and non-covalent interactions affect the chemical structure of proteins and phenolic compounds that also change their nutritional, functional, and biological characteristics (11).

## Factors Affecting Protein-Phenolic Interactions

The factors affecting protein-phenolic interactions are classified as internal factors (characteristics of proteins, types of phytochemicals, protein/phytochemical ratio) and external factors (temperature, pH, ionic strength, additional reagents, and other food components). The parameters that influence protein-phenolic interactions must be studied in order to investigate relevant applications.

### Internal Factors

#### Characteristics of Proteins

Dietary proteins' propensity to interact with phenolics is influenced by several factors, including their hydrophobicity, molecular weight (MW), conformational configurations, amino acid composition and amino acid sequence (9). According to strong hydrophobic interactions, hydrophobicity supports a strong binding between proteins and phenolics (18). Proteins with more basic amino acids and proline, as well as a bigger, more conformationally open and flexible structure, have a better chance of interacting with polyphenols (9). Furthermore, protein properties such as surface structure, total charges, and secondary structures have been found to have varying degrees of correlation with the non-covalent binding of dietary proteins to specific phenolic substances (9, 10).

#### Types of Phytochemicals

**Figure 1:**
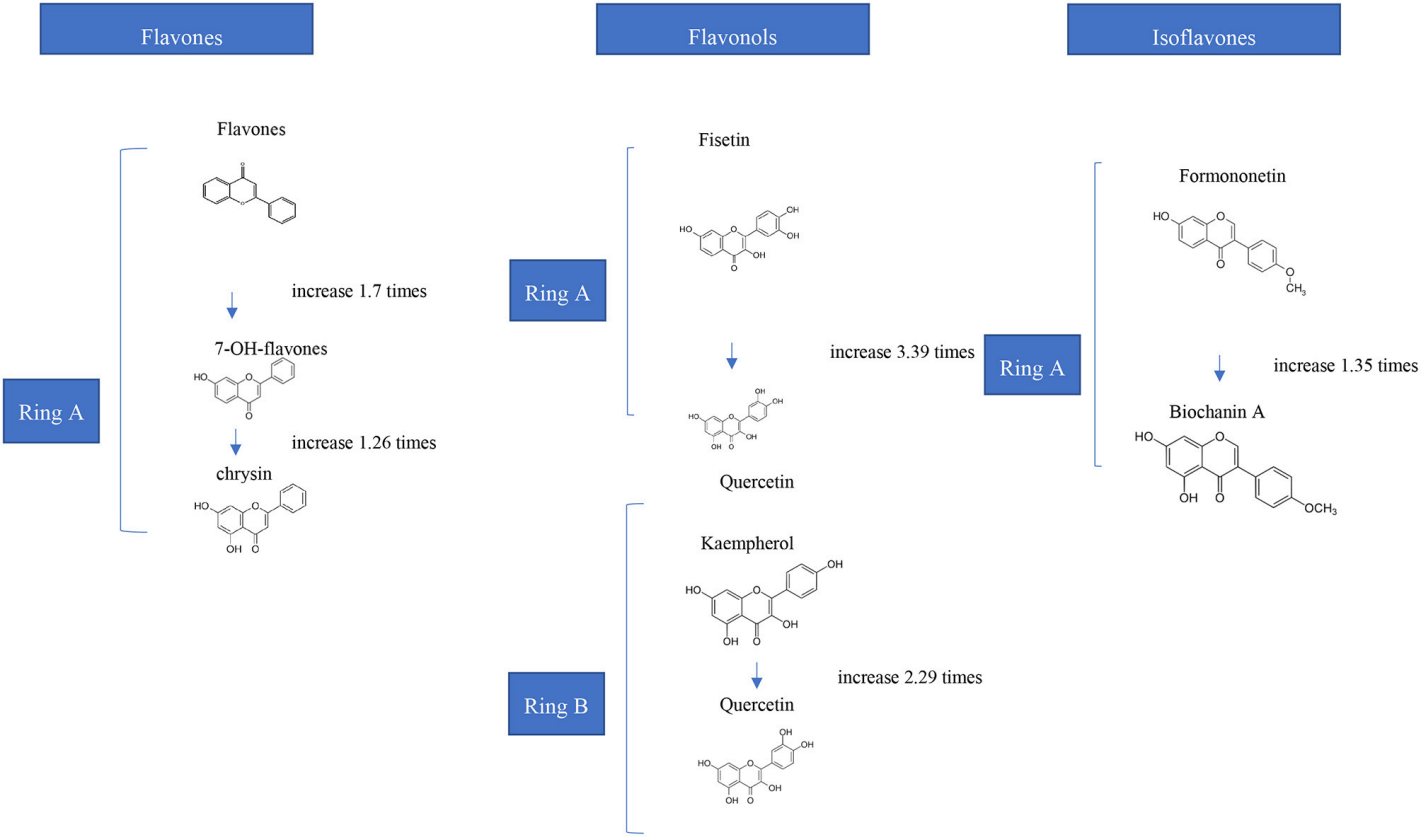
Effects of Hydroxylation of Flavonoids on the Affinities for Bovine Hemoglobin *in vitro* (24).

Occurrence of functional groups such as glycosyl, hydroxyl and methyl groups, as well as molecular weight, hydrophobicity, structural flexibility are the key determinants impacting phenolics' binding capabilities (19). It was reported that high molecular weight polyphenols in tea exhibited a stronger prosperity for interaction with milk proteins attributed to the fact that bigger polyphenols having more binding sites (20). Besides, it was reported in several studies that binding efficiency of tea catechins ((-)-epigallocatechin gallate (EGCG) > epicatechin gallate > epicatechin > catechin) to β-lactoglobulin was related with their molecular size (21, 22). Molecular flexibility of tannins also increase the interactions and binding bonds on proteins (23). In general, it was also reported in several studies that while hydroxylation of flavonoids at ring A and B (Figure 1) increase their interactions to whole milk proteins, methoxylation and methylation decrease the binding affinity and strength (24). Depending on sugar moieties and conjugation sites glycosylation also affect the interaction of phenolics with γ-globulin and hemoglobin. Moreover, hydrogenation of double bond between C2 and C3 also lower the binding affinity of phenolics (19, 24). By the way, hydroxylation of phenolic acids at the 3-position increases their binding affinity to BSA. However, their hydroxylation at the 2-/4-positions decrease their binding affinity. Individually, for increasing the binding strength, the hydroxy groups can be replaced with methoxy groups (4-position) or methyl groups (3-position) (25). Seczyk et al. (26) studied the interactions of pure phenolic compounds (gallic acid, ferulic acid, chlorogenic acid, quercetin, apigenin, and catechin) and phenolics from plant extracts including green tea and green coffee, with protein fractions including albumins and globulins of white bean. They have measured the physicochemical properties of complexes. Their results showed that, in most cases, phenolic type significantly affected the protein-phenolic interactions and measured properties (26).

#### Protein/Phenolic Ratio

Protein/phenolic ratio is another important factor that could affect the interactions between phenolic compounds and proteins. Multidentate and monodentate mechanisms can be involved at differing protein/phenolic ratio (27). The multi-dentate process requires a lower phenolic/protein ratio because phenolic chemicals operate as multi-site ligands by binding with several protein molecules or sites. In order to achieve cross linking of proteins to produce dimers or oligomers, phenolics should have large size. In the monodentate process, many phenolics bind with a single protein molecule, necessitating a significantly greater phenolic concentration (11). In complex phenolic combinations, synergistic or antagonistic actions between phenolics toward protein binding might occur, which should be taken into account when working with complex food matrices (28).

### External Factors

#### Temperature

Temperature can alter interactions by changing protein structures, ligand solubility, and the strength of certain non-covalent linkages. Thermal denaturation of proteins has been shown to affect phenolics' binding affinities to proteins on both sides: (1) by exposing previously buried hydrophobic sites by protein unfolding, and on the other hand, (2) by reducing interactions by forming aggregates with limited surface area (29, 30). Thermal modification of protein-phenolic interactions can result in structures due to phenolic oxidation into semiquinone and quinone intermediates (31). The binding affinities of proteins can be measured using fluorescence quenching analysis and It is possible to discern the key driving forces (9). Acknowledging the temperature-dependent relationships of such protein-phenolic combinations might aid in the development of functional food products.

#### pH

Protein conformational structures, as well as the binding properties of phenolics, may be significantly changed by pH. The tendency of phenolics, such as those found in berries and tea, to precipitate proteins has been shown to be pH-sensitive, especially at the isoelectric points of proteins, indicating that hydrophobic forces are involved in the interactions (32). Due to various reduced binding sites produced by protein secondary structural changes, increasing the pH from acidic to neutral values lowered the affinities of ferulic and chlorogenic acid to BSA (33). Abdollahi et al. (34) studied the interactions between β-lactoglobulin, and ferulic acid at ambient temperature temperature in relation to the dimer, and monomer forms of the protein at pH 7.3 and 2.4, respectively. They have reported that pH affects the binding site and strength of ferulic acid – β-lactoglobulin interaction and binding affinity was found to be higher when β-lactoglobulin was in monomer form (34). Non-covalent binding of dietary polyphenol extracts (tea, coffee, and cocoa) to β-lactoglobulin has been observed to alter with rising pH, probably due to differently charged states of constitutive components at different pH (35). Since the phenolics were auto-oxidized to quinones/semiquinones, which might interact with protein side-chain groups through covalent bonds, further rising the pH to alkaline conditions with oxygen would form covalent protein-phenolic complexes (13, 14, 36).

Moreover, Seczyk et al. (26) studied the interactions of pure phenolic compounds (gallic acid, ferulic acid, chlorogenic acid, quercetin, apigenin, and catechin) and phenolics from plant extracts including green tea and green coffee, with protein fractions including albumins and globulins of white bean, also determining the effect of ionic strength and pH on protein-phenolic interactions. It was reported that protein-phenolic interactions were affected by pH according to the results of relative protein solubility. The relative protein solubility was increased gradually as alkalinity increased from pH 5.0 to pH 11.0 (26).

#### Other Factors (Ionic Strength, Additional Reagents, Other Food Components)

Salt concentration in food products also affects the protein-phenolic interactions as it increases the ionic strength. The binding affinities of quercetin to BSA were reduced when ionic strength was raised by adding NaCl, probably due to a stronger hydration hull of the proteins with dissolved electrostatic connections (33). On the other hand, increased ionic strength in a buffering system improved the interactions between chlorogenic acid and BSA, which were largely mediated by hydrophobic forces (37). Furthermore, binding of EGCG to β-lactoglobulin in the presence of CaCl_2_ resulted in a bigger network of electrostatic calcium–EGCG bridging, resulting in greater levels of EGCG remaining in the complexes (38). Moreover, the increase of phenolic oxidase or free radicals such as OH radicals affect protein-phenolic interactions through oxidation of phenolics with irreversible covalent bonds. On the other hand, due to the suppression of enzyme activity, reducing substances such as Na_2_SO_3_ and ascorbic acid significantly decreased the oxidase-mediated connections (15, 39). Additionally, other macro nutrients including carbohydrates and fats also interact with proteins, however their potential for interfering with dietary protein-phenolic interactions is rarely examined (8).

## Implications of Dietary Protein-Phenolic Interactions

In general, it is considered that the reciprocal interactions in between food phenolics and proteins had a negative effect on the desired properties of both compounds such as functional properties and/or bioavailabilities (40), yet the improvements of varying techniques and approaches in recent years led to opposite results -in terms of boosted functional properties of proteins and bioavailability/antioxidant capacities of phenolic compounds- might also be possible and strictly dependent on the types of the compounds (5).

**Figure 2:**
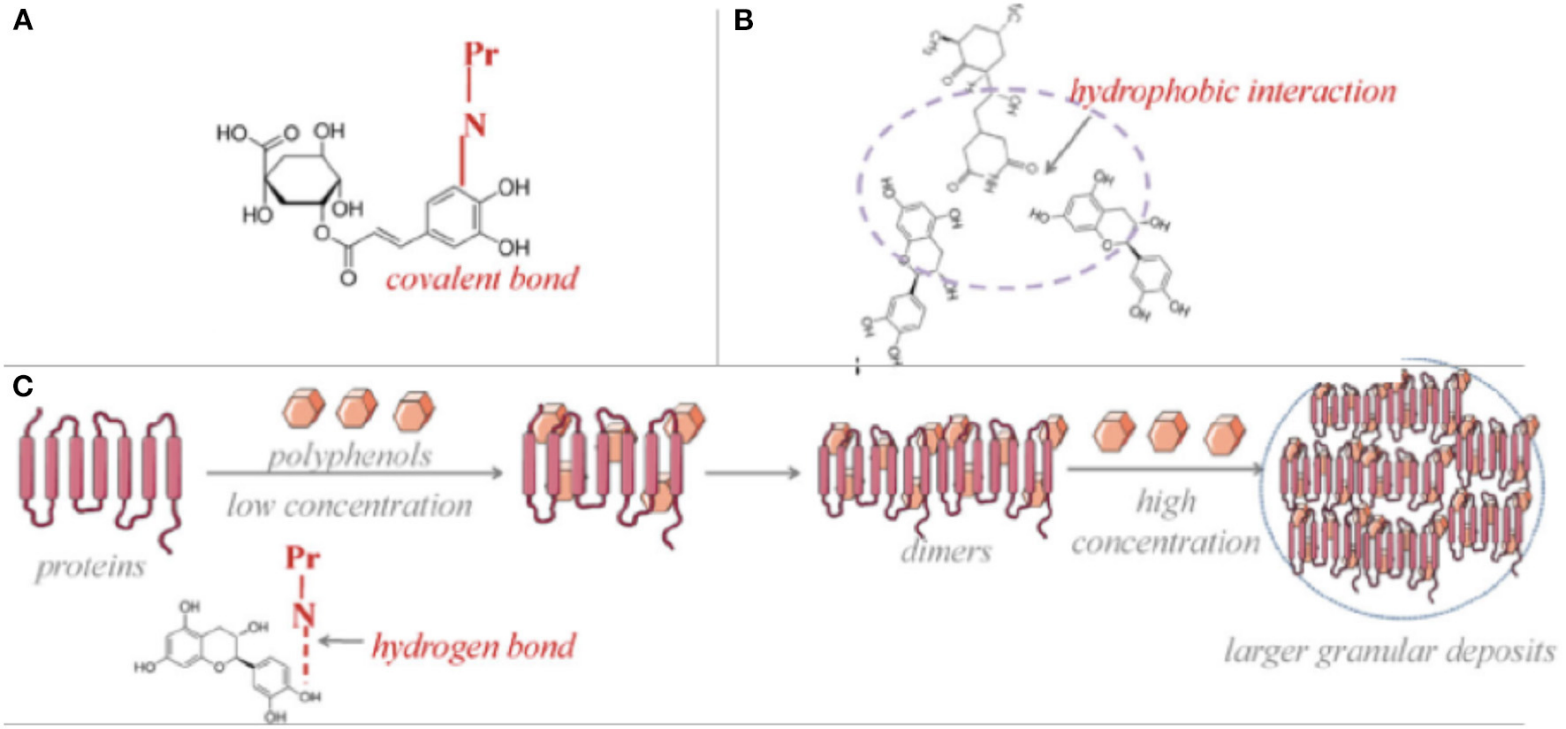
Mechanisms of protein-phenolic compounds interactions as conjugation via covalent bond **(A)**, complexation via hydrophilic interactions **(B)** and hydrogen bonds **(C)** [Adapted from (113)].

Dietary phenolics and proteins might interact following different mechanisms, which are dependent on the type of protein and phenolic compound, composition of food matrix, processing conditions as well as the digestion (9). Those interactions (Figure 2) were revealed having either covalent bonds as conjugates (chemical bonds) (Figure 2A) or non-covalent bonds as complexes (physical bonds), which have varying effects on techno-functional properties of the compounds but particularly the health promoting properties of both phenolic compounds and the food proteins. As explained in the previous part, food protein-phenolic conjugates occur either with free radical induction or under alkaline conditions while protein-phenolic complexes might comprise even as a result of a forced turbulence such as mixing/vortex action (41). Conjugates-covalent bonds mostly have resistant and irreversible interactions in between the molecules, on the other hand the complexes-non-covalent bonds are comprised of reversible hydrophobic interactions (Figure 2B), hydrogen bonds (Figure 2C), as well as ionic bonds which indicate that both types of food protein- phenolic compounds interaction might easily be generated by food processing and digestion periods. However, very few of research in the literature provided an insight about the type of protein-phenolic interaction mechanism in the matrix and most of the studies rather prefer to focus on the reciprocal consequences of the interactions, instead of the mechanisms. Depending on the prevalence of non-covalent interactions compared with the covalent bond formations during food processing and/or throughout the digestive tract after consumption, all reported protein-phenolic interactions in the food systems are assumed as “complexes,” unless other specified (9, 17). Consequently, the interaction in between the protein-phenolic compounds in food matrices are dynamic processes lasting until the digestion is completed and resulting distinctive indirect health promoting activities such as improved antioxidant capacities, *in vivo*/*in vitro* bioavailabilities for phenolics and better digestibility and nutritional levels for proteins. Furthermore, it was critically reviewed in a recent study that multistep interactions in between the ingested phenolic compounds and further derived functional proteins in the body such as enzymes, transporters, receptors and transcription factors are also matter of facts (42). Engineering the phenolic compounds as well as proteins to boost their varying range of health-attributed properties with increased retentions/stabilities are considered as one of the major factors to design and produce novel functional food products (43).

Since the stability and bioaccessibility of phenolic compounds were induced with protein interactions, phenolic compounds protect their antioxidative properties for longer times, with being less affected by external processing/environmental conditions and/or digestion periods (44). This protection effect was considered to increase the antioxidative potential of phenolic compounds indirectly throughout the whole digestion process. In this section, the latest studies covering the flavonoid and phenolic acid interactions with food proteins are reviewed for a positive health effect point of view, but the focus is mainly on the improved antioxidant activities. Only very recent papers (in the last 2 years) are covered to present the most novel outcomes.

### Flavonoid-Protein Interactions

Covalently structured soy protein isolates and black rice anthocyanins were exposed to *in vitro* gastrointestinal digestion, and it was indicated that the conjugated protein-anthocyanin structure had a higher degree of hydrolysis by around 20%, increased antioxidant capacity by 30 nmol/mg by DPPH assay and 1.5 nmol/mg by ABTS^+.^ assay (Trolox equivalent) compared to the control sample, however a decrease by 13% was reported for transepithelial transport of peptides across Caco-2 cell monolayer (45). Complex and conjugate forms of soybean protein isolate (5 mg/ml) and EGCG (0.2 mM) were investigated for the differences of protein digestibility as well as the antioxidant capacities following the *in vitro* digestion. It was observed that the protein digestibility of non-covalent complexes decreased to 59.14-65.71% compared with the control which was also higher than that of the covalent conjugates. Likewise, antioxidant activities by both ABTS^+.^ and FRAP assays were greater than the control sample in varying ranges for all stages of digestions for both interactions however, ABTS^+.^ trend was consistent for non-covalent structure with a promising scavenging activity increase from 29.02 to 360.51 μg Trolox/g protein (46). In another study with a similar approach, soybean protein isolate-EGCG complexes were used to fabricate alginate hydrogel beads in order to obtain higher antioxidant activities from the core material during the digestion process. It was indicated that 2:1 soy protein:EGCG covalent compounds enabled to produce 20% more stable and highly bioaccessible alginate beads by 20%, while non-covalent complexes enabled this ratio as around 10% (47). On the other hand, lentil protein isolates conjugated with quercetin were indicated in another study as to increase the radical scavenging activity with DPPH and FRAP assays by 66 and 46%, compared to the phenolic alone, respectively (48).

In a study mainly focusing on the polyphenol stability during a storage period (12 days at 37°C), strawberry polyphenol extract (dominating by flavonols and phenolic acids) was used to make non-covalent complexes with canola protein extract. Degradation rate of the compounds such as pelargonidin-3-*O*-glucoside, cyanidin-3-*O*-glucoside, pelargonidin-3-*O*-rutinoside, pelargonidin-3-*O*-malonylglucoside, kaempferol-3-*O*-malonylglucoside were found to decrease significantly in a range of 17–44%. However, no significant difference was observed for total antioxidant activity of defined phenolic compounds after complexation with canola protein extract (49).

Bioaccessibility of coffee beverage phenolics was observed to increase by applying different protein sources as skimmed milk or soy proteins. Total bioaccessibility of coffee phenolics increased with skimmed milk and soy protein application by 37.01–64.21% and 24.74–47.32%, respectively while individual phenolics bioaccessibility *in vitro* increased by 4.40–27.29% and 12.02–28.61% under heat treatment (25, 90, 121°C). An antioxidant activity analysis was not conducted however, the study was observed to have the potential to increase the antioxidative properties (50).

Besides the antioxidative property improvement potentials of the phenolic interactions with food proteins, their capability to decrease the allergen effects of particular food proteins is another research interest of protein-phenolic interaction, with a growing attention. The potential of covalent and non-covalent interactions between the major milk proteins (α-casein and β-lactoglobulin) and cyanidin-3-*O*-glucoside were investigated aiming to reduce/mask the allergenic activity of milk proteins. Covalent conjugated forms significantly reduced the digestibility of milk proteins (a decrease range 5–11% with varying phenolic:protein ratios as 20, 30, 50) for both proteins, while showing lower IgE binding properties compared with the non-covalent complex forms as a decrease by 54 and 88% for α-casein and β-lactoglobulin conjugates (when the phenolic:protein ratio is 50), respectively (16). Similar promising results were presented by Pu et al. (51) who intended to examine the allergen reduction potentials of six different flavonoids such as EGCG, naringenin, quercetin, kaempferol, myricetin, and phloretin non-covalent complexations with β-lactoglobulin. Molar ratios of protein and phenolics were applied as 1:1–1:5. All phenolic complexations were indicated to perform antigenic inhibition for β-lactoglobulin in a descending order; 72.6, 68.4, 59.7, 52.3, 51.4, and 40.8% for EGCG, phloretin, naringenin, myricetin, kaempferol, and quercetin (51). Due to the promising results of existing antigenic factor inhibition studies for varying food proteins with distinctive types of phenolic compounds, the research interest on this area has been growing (52).

### Phenolic Acid-Protein Interactions

According to the literature, it is a very well know issue that the phenolic acid interactions with varying food proteins have a significant potential to increase their antioxidant activities as well as improvements in many other reciprocal techno-functional properties. For instance, the non-covalent interactions of whey protein isolate and casein with chlorogenic acid (240 μmol/g protein) were evaluated in terms of the interaction effect on the protein digestibility. It was observed that at the end of 2 h, degree of hydrolysis was increased by 7.7 and 5.3% for casein and whey protein isolate, respectively compared with the samples without phenolic interactions. The same samples also yielded a significant antioxidant capacity increase by 460 and 400% for casein and whey protein isolate, respectively based on the ABTS^+.^ assay (53). A different study focused on the type of complexations in β-lactoglobulin (150 μM) and chlorogenic acid (1.5 mM) under different thermal conditions as well as the alteration of antioxidant capacities. It was indicated that, with the temperatures below 60°C, the interactions were observed as non-covalent however, above that temperature covalent interactions started to be established. Both interactions retarded the loss of antioxidant capacities based on ABTS and FRAP assays. Non-covalent complexations had no significant increase on antioxidant capacity by ABTS assay, however covalent conjugates showed increased capacities (the highest increase as 56.18% at 121°C). Based on FRAP assay, both interactions increased the antioxidant capacities but the highest one (23.36%) was observed for 85°C conjugates (54).

In another study, covalent crosslinking effect of soy protein isolate (12%, w/v) on the antioxidant capacity of tannic acid (29, 58, 88, 117, 146 μmol/g protein) was investigated with DPPH, FRAP, and ABTS^+.^ assays. It was indicated that 146 μmol/g protein tannic acids yielded the highest antioxidant capacity in terms of all three assays in a pH (9.0–11.0) dependent manner (55). Soy protein isolate was covalently interacted with chlorogenic acid (20, 40, 60, 80, 100 μmol/g protein) and based on the ABTS and DPPH assays, an antioxidant capacity increase by 10.87% and 33.08 μmol Trolox/g protein was observed for the chlorogenic acid concentration of 100 μmol/g protein (56). Another plant-based material, pea protein isolate was studied in case of non-covalent complexations with chlorogenic acid (50 μmol/g protein). The main interaction in between the phenolic acid and protein sample was declared as electrostatic interactions. Degree of hydrolysis as an indicator for *in vitro* digestion for pea protein was found to increase by around 5% (57).

Consequently, protein-phenolic interactions are of significant techno-functional reactions ending up with crucial biological activities such as significant antioxidative properties, varying dependent on the types of the compounds, processing conditions and physical media. However, further in-depth research is still required for better tailoring and designing for novel functional foods with boosted health promoting factors.

## Implications of Dietary Polyphenols-Viral Protein Interactions

In recent years, the pandemic of viral diseases caused serious economic losses and community associated infections, which forced the scientific community to investigate less toxic antiviral phytomolecules instead of using nucleic acid analogs, protease inhibitors or other toxic synthetic molecules as antiviral therapeutics. Although the use of natural antimicrobial agents for food preservation is a trend that is followed by both consumers and food manufacturers, the studies on the antimicrobial activity of phenolic–protein conjugates are very limited (58). On the other hand, there are few studies on the antibacterial activity of protein-phenolic conjugates (59, 60), and the antiviral mechanisms of the conjugates are still unclear until now. The existing studies have focused on the antiviral effect of phytochemicals alone (7). Most of the studied plant-derived secondary metabolites were polyphenolic compounds. They have three main modes of action in treating/preventing viral diseases.

(1) They can bind to the various non-structural proteins (enzymes) to replicate the virus itself, thus inhibit early and late phase of viral replication.
(2) They can bind to structural protein (spike protein) of virus itself, thus inhibit the virus from binding and penetration to the host cells.
(3) They can bind to cell receptor proteins and inhibit virus penetration into the cell.

Since antiviral mechanism of food protein-phenolic conjugates is not clear with the existing literature, we focused on the viral protein-phenolic interactions in this review to better understand the chemistry of protein-phenolic binding.

In the literature there are many natural product-derived phytochemicals investigated as potential agents against viruses. The majority have been classified as polyphenols among the most promising small molecules identified as virus inhibitors. Most studies interpret whether phytochemicals have an antiviral effect based on changes in virus numbers, but they do not show that this antiviral effect is related to the binding between phytochemical and virus protein. We have not included the details on such studies investigating the antiviral activity of numerous phytochemicals in this review. Rather, our aim is to explain the antiviral effect of polyphenols by focusing on their binding properties with viral proteins through the most studied viruses in the literature.

Recently, molecular docking studies have become popular and provided detailed information about ligand-protein binding properties. According to the results, protein-phenolic interactions have direct effect on viral inhibition. These interactions generally occur between phytochemical and non-structural proteins of the viruses, but binding with structural viral protein was also available though.

### Corona Viruses

Novel corona-virus (SARS-CoV-2 or COVID-19) has structural and non-structural proteins, which are the key viral molecules, involved in attachment, replication and reproduction of viral particle in the human host cells. SARS-CoV spike glycoprotein (S protein) is the surface protein (structural protein) that is mainly responsible for the initial attachment with the host cells receptor, Angiotensin-converting enzyme (ACE2) (61). SARS-CoV-2 chymotrypsin-like main protease (3CLpro or Mpro, corresponding to Nsp5), papain like protease (PLpro, a domain within Nsp3) are non-structural viral proteins which facilitates viral assembly by cleaving polyproteins (62). RNA dependent RNA polymerase (RdRp) is another nonstructural protein that is vital for viral life cycle (63). These viral protein molecules serve as a novel target to inhibit the viral lifecycle in human host cells.

Basu et al. (64) mentioned that the phytochemical hesperidin (flavonoid) can bind to ACE2 protein. It can also interact with the bound structure of ACE2 protein and spike protein of SARS-CoV2 (64). The binding sites of ACE2 protein for spike protein and hesperidin, are located in different parts of ACE2 protein. Molecular dynamics and docking studies confirmed the conformational change in three-dimensional structure of protein ACE2 due to ligand spike protein. This compound modulates the binding energy of bound structure of ACE2 and spike protein. After all, in the presence of hesperidin, the bound structure of ACE2 and spike protein fragment becomes unstable and lost its viral activity in SARS-CoV-2 infection.

Moreover, natural flavonoids, quercetin, epigallocatechin gallate and gallocatechin gallate (GCG) showed good inhibition properties by binding to the 3CLpro active site and the 3-OH galooyl group, which was required for SARS-CoV inhibitory activity (65). Green tea polyphenols also have SARS-CoV-2 interaction with catalytic residues of major protease (Mpro). Green tea EGCG binds to spike protein by having the highest affinity to SARS-CoV-2 (66). Catechin is another possible therapeutic phenolic compound able to bind to the viral spike protein and ACE2 of the host (67).

**Figure 3:**
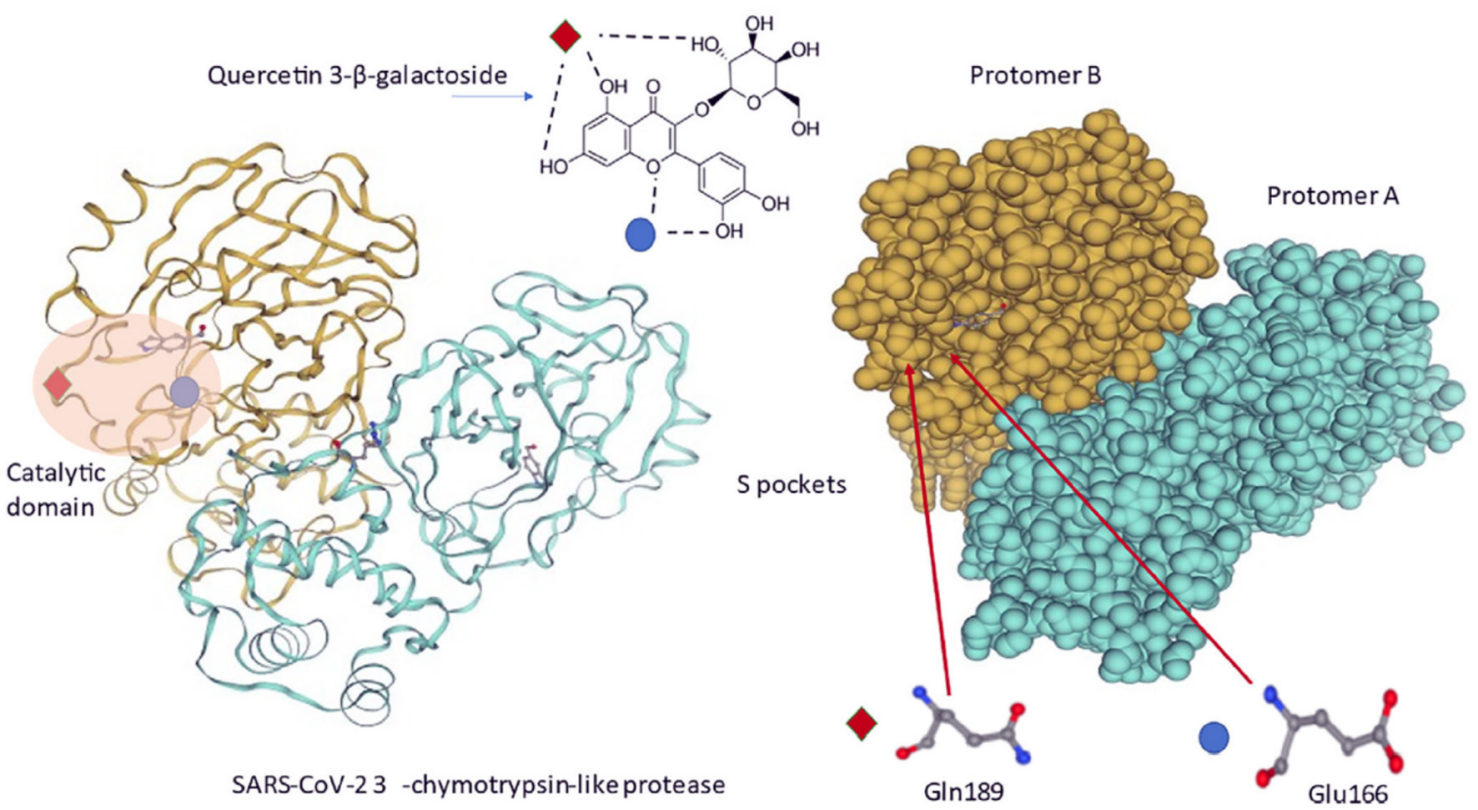
Molecular interactions between quercetin-3-β-galactoside and SARS-CoV-2 3CLpro. Quercetin-3-β-galactoside forms hydrogen bonds specifically with Gln189 and Glu166 amino acids located inside a specific pocket hollowed in 3CLpro surface (70).

In another study, binding of flavonoids, herbacetin, isobavachalcone, quercetin 3-β-d-glucoside and helichristetine was studied by the fluorescence spectroscopy. As a result, flavonols were found to bind to the Middle East Respiratory Syndrome Coronavirus (MERS-CoV) 3CLpro catalytic site and inhibit the virus (68). Chen et al. (69) studied the binding of quercetin-3-β-galactoside to SARS-CoV 3CLpro and found its protease inhibition role (69). Di Petrillo et al. (70) have schematized the interaction between quercetin-3-β-galactoside and the SARS-CoV-2 protease in Figure 3 (70).

The interaction of flavonoids presents in *Galla chinensis* extract to the structural protein of SARS-CoV was studied by Wang and Liu (71). They observed that the flavonoids could bind to the surface of the spike protein of the virus and prevent their penetration to the cell (71). A recent study with phytochemicals used for the inhibition of SARS-CoV-2 Mpro enzyme indicated that the flavonoid, 5,7-dimethoxyflavanone-4-*O*-β-d-glucopyranoside, interacts with the target enzyme and this interaction is strengthened by the establishment of five hydrogen bonds with a contribution of hydrophobic interaction, too (72). A flavonolignan, silybin (73) and a phenol phytocompound, 6-gingerol (74) have exhibited higher binding affinity and hydrogen bond interaction with non-structural protein targets in SARS-CoV-2 in comparison to currently used repurposed drugs against SARS-CoV-2. Gingerol also exhibited good binding affinity with SARS-CoV-2 spike glycoprotein. Gingerol formed hydrogen-bonded interaction with those viral proteins but with different amino acid residues. There was also a hydrophobic interaction between them (74). The electronic distribution (DFT study) also provided a clear picture of SARS CoV-2 protein-gingerol interactions and supported the molecular docking results.

Glycyrrhiza (liquorice) active compounds against spike (glycoprotein) and non-structural protein of SARS-CoV-2 were investigated (17, 75). Glyasperin A (flavonoid) had a high interaction with endoribonuclease (NS protein) and a high binding ability with the protein receptor cavity. A covalent binding of luteolin-7-*O*-glucuronide and chlorogenic acid (phenol) present in *O. sanctum* to Cys145 of Mpro of SARS-CoV-2 have been mentioned *in silico* analysis, so this may hinder the virus enzymes (76). Kumar et al. (77) recently reported novel natural metabolites namely, ursolic acid, carvacrol and oleanolic acid as the potential inhibitors against main protease (Mpro) of SARS-CoV-2 by using integrated molecular modeling approaches. They found that three ligands were bound to protease and these chemical molecules had stable and favorable energies causing strong binding with binding site of Mpro protein (77).

**Figure 4:**
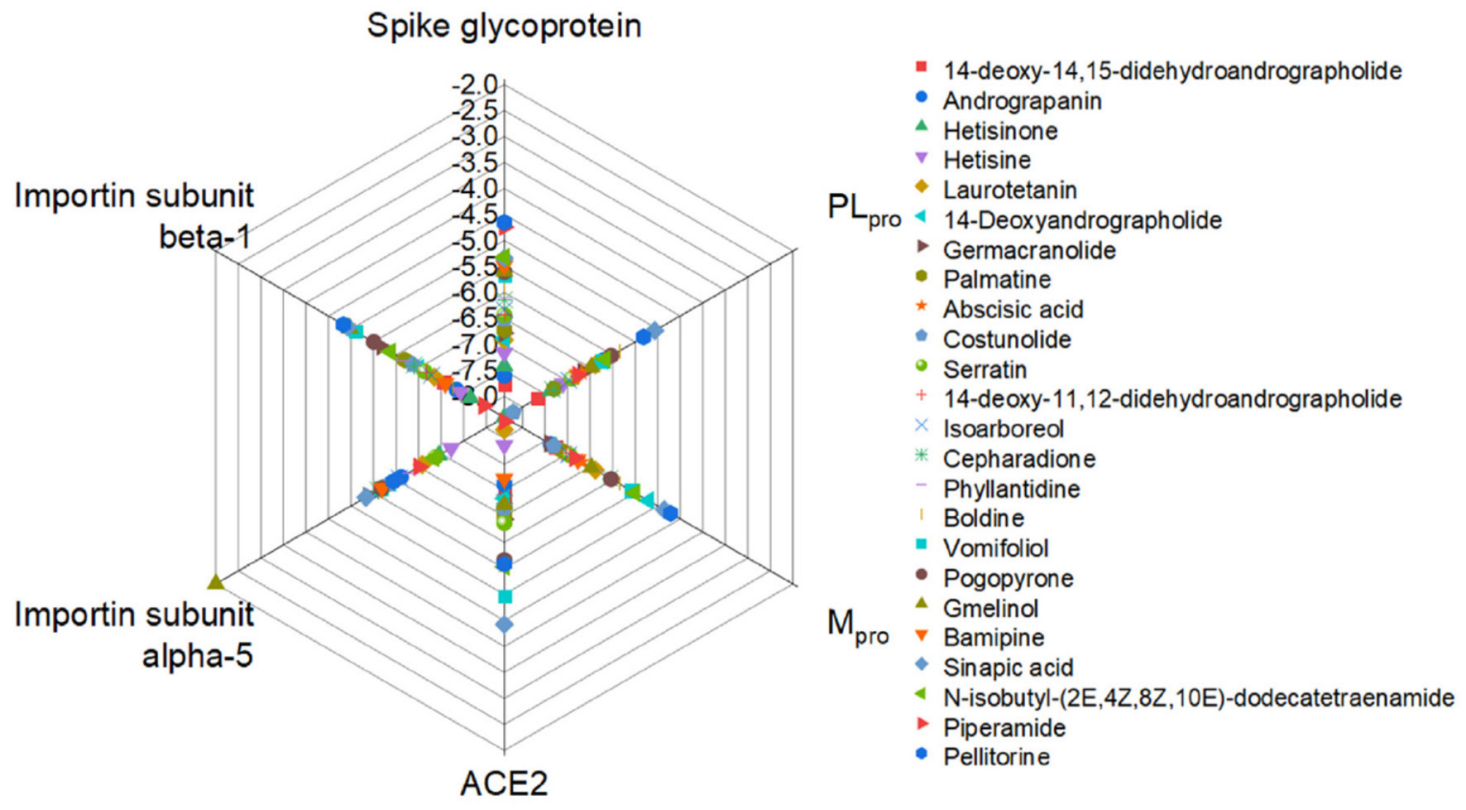
Relative binding energies of phytochemicals with the viral and host proteins (78).

Lastly, Singh et al. (78) analyzed the binding affinity of 586 phytochemicals to the viral proteins (glycoprotein spike, Papain-protease, and protease main) and host proteins (ACE2, Importin-subunit a-5, and b-1). Their hydrogen bonds and hydrophobic interaction, as well as the binding energies and interactive amino acid residues, were categorized. Figure 4 shows the binding energy of the most potent phytochemicals. Hetisinone (alkaloid) showed the highest binding energy among all selected phytochemicals. Hetisinone formed 6 H-bonds whereas, control drug, hydroxychloroquine (HCQ), formed 4 H-bonds. This means that the interaction by hydrogen bonds between hetisinone and ACE2 represented a strong interaction than the control drug molecule. The viral-host interaction was disturbed by HCQ binding to the ACE2 receptor (allosteric region), which can inhibit the viral penetration to the human cell (78).

### Hepatitis Virus

Manvar et al. (79) identified the anti-Hepatitis C virus (HCV) activity of four different compounds obtained from crude extract of *Eclipe alba*. These are 3,4-dihydroxybenzoic acid (1), 4-hydroxybenzoic acid (2), luteolin (3), wedelolactone (4) and apigenin (5). They used fluorescence spectroscopy technique and quenching the fluorescence emission of the non-structural HCV protein NS5B (an RNA-dependent RNA polymerase) indicating their ability to bind with the enzyme. In addition to the fluorescence quenching technique, they used cell culture system and found that compounds 3, 4 and 5 bound to the enzyme HCV replicase which resulted with the inhibition of HCV replication (79). In fact, the results showed that these compounds have a dose-dependent synergistic inhibitory effect on the enzyme activity, which confirms that HCV replicase have five different binding sites for small molecules that might bind and block enzyme activity (80). The observed synergistic or additive effect of these compounds against HCV replication suggesting that these compounds are more beneficial when used together in the crude extract (79).

Recently, the possible interaction of quercetin with HCV's non-structural protein was identified *in vitro* (70). Molecular docking study of Zhong et al. (81) provided that quercetin derivates can interact with NS5B (non-structural protein 5B in HCV) by two magnesium ions as well as establish coordination with residues at the active site (81). Another molecular docking study showed that quercetin can bind and inhibit the NS2 (non-structural protein 2) protease of HCV (82). Its interacting ability with different proteases causes virus replication blocking and reduces HCV-induced reactive oxygen and nitrogen species (ROS/RNS) generation in replicating cells.

Furthermore, quercetin extracted from *Guiera senegalensis* demonstrated a high anti-Hepatitis B virus (HBV) potential. Data showed that quercetin binds the active site of HBV Polymerase by forming nine hydrogen bonds that stabilizes the quercetin-polymerase complex with an estimated free energy of 7.4 kcal/mol (83). These interactions have important contributions for stabilization of the complexes. Another research suggested that quercetin and its derivatives exert their action by interacting with the M2-1 protein, involved in genome replication and transcription by forming the complex RNA-dependent RNA polymerase (84). Caffeic acid was also found to inhibit HBV by binding to non-structural proteins of the virus (85).

### Dengue Virus

Dengue, a mosquito-borne disease, has appeared as a major infectious disease globally. In the study of ul Qamar et al. (86) different phytochemicals were used against the non-structural protein of Dengue virus (DENV), which is the NS2B/NS3 protease having the ability to cleave viral proteins (86). As a phytochemical source, garcinia is a genus of flowering plants in the family *Clustaceae* native to Asia, America, Australia, tropical and southern Africa, and Polynesia. With the help of molecular docking, (-)-gossypol (polyphenol), mangostenone C (prenylated xanthones), garcidepsidone A (aromatic), 4-hydroxyacetophenone4-*O*-(6'-*O*-beta-D-apiofuranosyl)-beta-D-glucopyranoside, demethylcalabaxanthone (flavone glycosides), and mangostanin (natural xanthonoid) were found to be bound deeply inside the active site of DENV NS2B/NS3 protease. They had hydrophobic interactions with catalytic triad of the protease enzyme. Thus, it can be concluded from the study that these gracinia phytochemicals could serve as important inhibitors to inhibit the viral replication inside the host cell.

**Figure 5:**
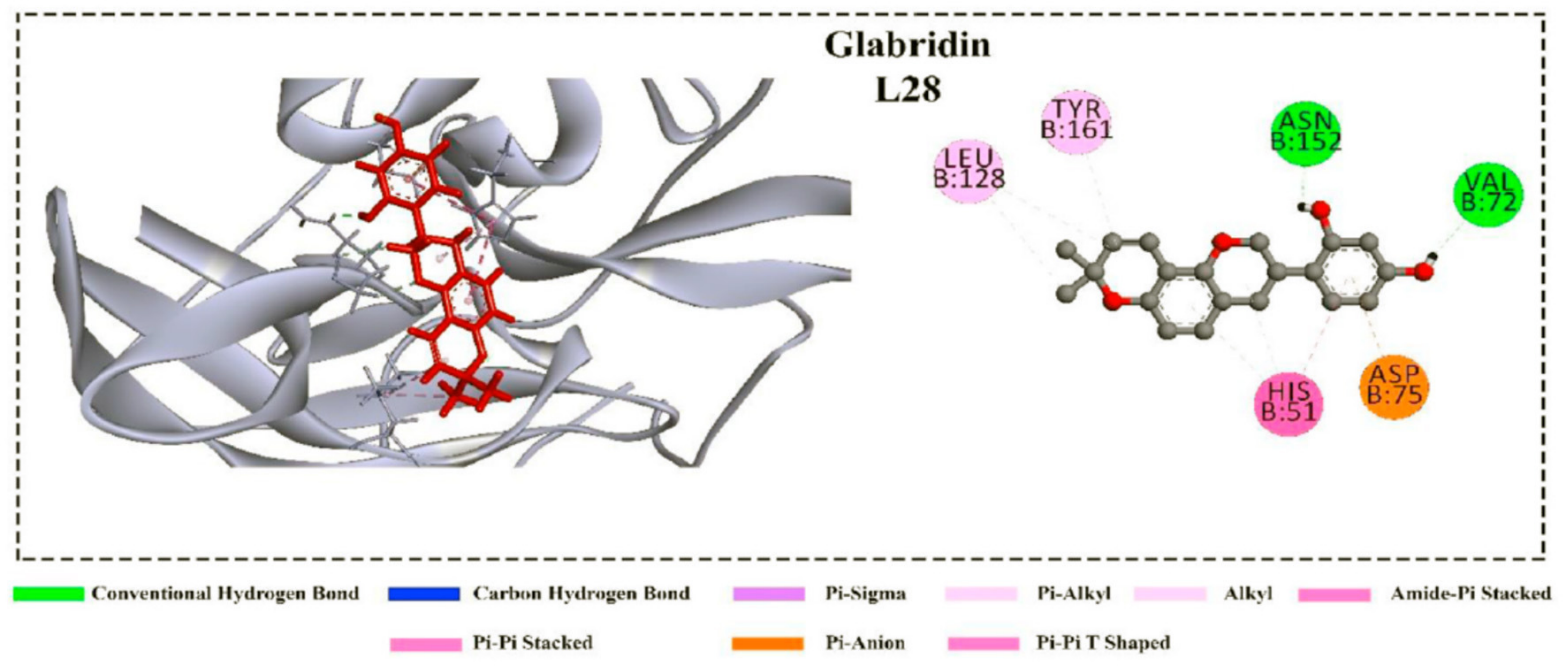
Non-covalent interactions of the glabridin with the NS3 protease (87).

In addition, cyanidin 3-glucoside, dithymoquinone, and glabridin were predicted to be potent inhibitors against the NS3 protease according to their binding affinity. These ligands showed several non-covalent interactions, including hydrogen bond, hydrophobic interaction, electrostatic interaction, pi-sulfur interactions (Figure 5). After interaction, the C terminal region of NS3 tends to form a helical structure. This deviation from the initial structure resulted in enzyme inhibition (87).

In the other study, kushenol W and kushenol K flavonoids showed not only strong hydrogen binding but also hydrophobic and non-covalent interactions with structurally and functionally active site of dengue proteases (88). At last, kushenol W and kushenol K disrupted non-structural DV protein functions crucial for viral replication, thereby inhibiting DENV infectivity. Moreover, baicalin has a good binding ability with the NS3/NS2B protein of DENV. Recently, Loaizo-cano et al. (6) reviewed 20 different phenolic compounds against DENV and their good binding affinity to viral particles and proteins but didn't mention the interaction mechanism and types of bindings (6).

### Other Viruses

Currently novaccines to prevent Zika virus (ZIKV) infection is available. In the work of Byler et al. (89), potential anti-Zika viral agents from 2263 plant-derived secondary metabolites have been investigated in *in-silico* molecular docking studies. Most of them were polyphenolic compounds (total 1043 of aurones, chalcones, chromones, coumarins, flavonoids, isoflavonoids, lignans, stilbenoids, xanthones, and miscellaneous phenolics). It was shown that the main antiviral molecule targets are several ZIKV non-structural proteins, typically the various enzymes to replicate the virus itself, such as NS2B-NS3 protease, helicase, NS5 methyltransferase. The best binding phytochemical ligands were the polyphenolics with generally two phenolic groups with flexible links to have strong connection to several of the protein targets. For example, rosemarinic acid, cimiphenol, and cimiracemate B showed relatively strong binding energies, thus they inhibit the replication of the virus. They also mentioned that the poorest enzyme binding ligands were found as terpenoids due to their small size, and their paucity of functional groups (89).

Many phenolic compounds have also been related to antiviral activity against many viruses, such as Herpes simplex virus (HSV), influenza virus (IV), RSV, measles, and rotavirus. These studies demonstrates that flavonoids could be one of the most active compounds against different types of viruses with multiple inhibition mechanisms, such as the inhibition of virus adsorption, entry, binding, and replication by binding both viral and host cell proteins (6). For example, quercetin showed high binding activity on cap-binding site of the PB2 (polymerase basic 2) of influenza viral RNA polymerase. Moreover, it could interact with influenza NA protein (virus surface glycoprotein neuraminidase) and block virus entry at the initial step (90). Flavonoids have non-covalent binding to reverse transcriptase and block RNA synthesis of HSV (7). EGCG also inhibits HSV by binding to their gB, gD, or other envelope proteins (91).

## Implications of Dietary Phenolic on Microbiota and Microbial Proteins

Human gastrointestinal tract (GIT) is the home for hundreds of different microorganism species and this microbiota plays crucial roles for healthy human homeostasis, including immune and digestive systems (92). The GIT microbiota is not homogenous but very variable through different part of the system; however, it is one of the most populated microbial habitats on the earth (93, 94). All the commensal microorganisms reside within the mucosal layer of the GIT, except pathogenic ones, which go into epithelial layer by digesting the mucosa (95). The GIT microbiota is shaped by the host factors, one of the most important is the diet (96). The members of GIT microbiota are able to metabolize sugars, especially the indigestible ones (97). Not only the sugars, but also they can metabolize other ingredients of the diet. For instance, dietary phenolic compounds can be transformed by GIT microbiota before absorption and this transformation can modulate their biological activities (98). Very small portion (5–10%) of these phenolic compounds taken with the foods are directly absorbed through the small intestine, while most are transported to the large intestine, especially colon, and there they are metabolized by the microbiota (99). However, the transformation of the phenolics is dependent on the nature of the phenolic compound, as well as the microbial species (100). For example, one of the most-studied and very-well known phenolic compounds, resveratrol, is metabolized by human GIT microbiota, shown both *in vitro* and *in vivo* (101). In the study of Bode et al., fecal samples of different volunteers were used to investigate *in vitro* metabolism of trans-resveratrol. They showed fecal samples completely degraded trans-resveratrol and then metabolites were detected after 2–24 h of fermentation. Furthermore, in the same study, a separate human *in vivo* study with 12 volunteers was conducted and trans-resveratrol was given orally to the volunteers. After 24 h, a subsequent proportion of trans-resveratrol was found to be metabolized by GIT microbiota and same metabolites found in *in vitro* study were also detected in the urine samples, which confirms that trans-resveratrol was metabolized into three metabolites, main ones being dihydroresveratrol and lunularin. This study also confirmed the interindividual differences of resveratrol metabolism by GIT microbiota as microbiota can differ between the individuals (101). A pure-culture study investigated the metabolism and transformation of different but related glycosylated phenolic compounds using *Lactobacillus acidophilus* NCFM (102). They showed that *Lactobacillus acidophilus* NCFM can use such phenolic compounds and other phytochemicals as carbon source. In this mentioned study, eleven plant glycosides, including salicin, rutin, vanillin, and arbutin, were used and esculin, fraxin, and salicin supplemented to the growth were completely utilized by the bacterium. Vanillin was almost completely utilized and the growth of the bacterium in these phytochemicals reached 0.3 to 1.3 (OD_600_) in 200-μl cultures in 96-well plates. Furthermore, it is very-well known that dietary phenolic compounds affect both physiology and gene expressions of gut microorganisms. Thus, these effects can result from interactions of phenolic compounds with microbial macromolecules, including proteins. As the phenolic compounds usually interact with food proteins or host proteins, these conjugated complexes should further affect the gut microbiota. Theilmann et al. used transcriptomics to examine which genes are affected by these phytochemicals. Genes related with carbohydrate metabolism were differentially regulated in the phytochemical-supplemented group, with respect to control in which glucose was used as carbon source. In addition, the genes encoding the proteins related with mucus, fibrinogen, and epithelial cell adhesion were also upregulated (102). These proteins may have roles in adhesion to the host components and this upregulation may lead to higher adhesion, which means better colonization in the GIT. Previously, it has been also showed that polyphenols affect the proteome profile of this bacterium, as well as its adhesion to mucus and host cells (103). These studies suggest that plant phenolic compounds can interact either directly to the probiotic proteins or indirectly by altering the gene expressions.

**Figure 6:**
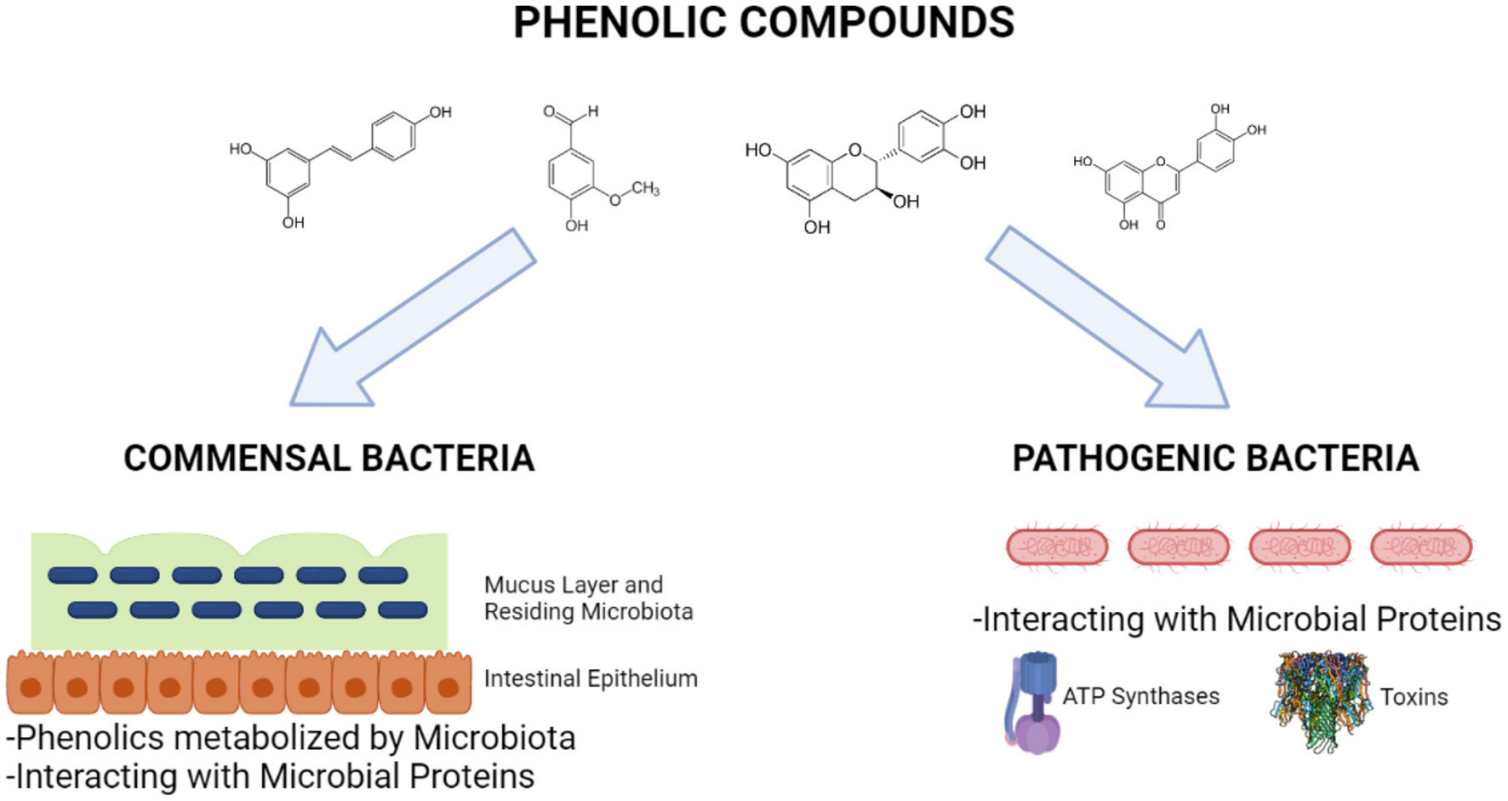
Interactions of phenolic compounds with commensal and pathogenic bacteria (Created with BioRender.com).

Phenolic compounds can interact not only commensal microorganisms residing in the GIT or probiotics but also pathogen microorganisms so that they exert antimicrobial activities (Figure 6). These compounds especially affect proteins of such organisms, thus inhibiting crucial proteins and microbial growth, leading to antimicrobial activity. The microbial proteins affected by phenolic compounds belong to membrane (efflux system, cell envelope metabolism, and ATP synthase system), anabolic and catabolic reactions, pathogenic and antibiotic-resistance mechanisms, such as toxins and virulence factors transporters and antibiotic-inactivating enzymes, virulence factors, biofilm, and DNA metabolisms. Bacterial cell membrane is a complex system that plays roles as barrier between outside and inside of the cell, regulator of the osmotic, energy, and lipid systems, as well as cell wall maintenance (104). The major antibiotics, including penicillins, cephalosporins, and polymixins, target the bacterial membrane and cell wall, thus disrupting their functions and leading to bacterial death. The studies showed that some phenolic compounds, especially flavonoids can inhibit the efflux pumps present in the bacterial membranes and having roles in drug modulation (105, 106). A study done by Sinsinwar et al. showed that catechin treatment of *Staphylococcus aureus* led to decreases in the activities of superoxide dismutase and catalase enzymes, which are responsible for anti-oxidant system of the bacteria (107).

Bacterial energetics is mainly driven by ATPase activity, which is a complex protein structures responsible for ATP production. Other than some known antibiotics or antibacterial compounds, phenolics can also be inhibiting agents of ATPase system. For example, resveratrol, piceatannol, quercitrin, and quercetin inhibit ATPase belonging to *Escherichia coli* but in different degrees (108). Among these, the ATPase activity was completely inhibited by piceatannol, thus can lead to bacterial death; however, this study was performed *in vitro* using purified ATPase or membrane. Furthermore, Chinnam et al. also investigated the inhibitory effect of flavonoids on *E. coli* ATPase (109). In their study, seventeen polyphenols were used and *E. coli* growth, as well as inhibitory effect on purified ATPase activity were investigated. They found polyphenols used in the study caused complete, partial, slight, or no inhibition on ATPase activity. For instance, morin, silymarin, baicalein, silibinin, epicatechin, rimantadine HCl, and amantidin completely inhibited the ATPase activity, while hesperidin, chrysin, kaempferol, diosmin, apigenin, genistein, and rutin led to partial inhibition. On the other hand, luteolin, daidzein, and galangin showed insignificant inhibition. The authors stated that presence of number of hydroxyl groups can be important for the inhibition (109).

Pathogenic bacteria produce virulence factors, which are various molecules promoting disease state by invading and attacking host cells. They include endotoxins (found within the cell) or exotoxin (released to the outside of the cells). Some phenolic compounds target and inhibit such virulence factors. For example, flavone and luteolin, which are flavonoids, were found to reduce production of staphyloxantin, a carotenoid pigment produced by some strains of *Staphylococcus aureus*, and acting as a virulence factor (110). Furthermore, flavone also inhibited gene expression of α-hemolysin, causing red blood cell lysis by disrupting the cell membrane, investigated using qRT-PCR technique (110). Another study done by Stockovic et al. showed that methanolic extract of *Phlomis fruticosa* L. (Jerusalem sage) inhibited the expression of staphyloxantin (111). Another common pathogen is *Listeria monocytogenes*, which produces a virulence factor called listeriolysin O. This bacterium causes gastroenteritis, meningitis, and abortions and listeriolysin allows this bacterium to escape phagocytosis by the host immune system and also disrupt the vacuole of the host cells. A study done by Wang et al. indicated that fisetin, a plant flavonol present in many plants including strawberries, apples, persimmons, onions and cucumbers, inhibits listeriolysin O–induced hemolysis, suggesting that fisetin antagonizes the hemolytic activity and protects the red blood cells by directly or indirectly interacting with the toxin (112). Furthermore, fisetin also facilitates the elimination of *L. monocytogenes* by macrophages by blocking bacterial escape into the cytosol. These studies implicated that those phenolic compounds inhibit the pathogenic bacteria by interacting with their proteins that are crucial for bacterial growth or pathogenesis. However, more molecular investigations are required to enlighten how exactly phenolic compounds interact and inhibit microbial proteins.

### Concluding Remarks

Phenolic compounds are abundant bioactive compounds in human diet and used as natural colorants and preservatives in foods. Despite the valuable biological activities of phenolics, such as antioxidant, antimicrobial, anticancer, antiallergenic, and anti-inflammatory effects, little is known about the health-related activity of phenolic-protein conjugates. Thus, in the last decade there is a growing interest on the functionalization of proteins with phenolic compounds. The interaction of food polyphenolics and proteins, as well as its effect on the functional properties including antioxidant and antimicrobial properties related to the chemistry of their binding, are reviewed in this paper.

Protein-phenolic interactions take place as covalent and non-covalent interactions. Protein interaction with polyphenolics-flavonoids and phenolic acids is influenced by their hydrophobicity, molecular weight, conformational configurations, amino acid composition and sequence. Type of phenolics has also significant effect on the protein-phenolic interactions and biological properties. However, very few studies in the literature provided an insight about the type of protein-polyphenolic interaction mechanism in the matrix and most of the studies focused only on the reciprocal consequences of the interactions, instead of the mechanisms. These reciprocal interactions between proteins and phenolics result in a change in the nutritional, functional, biological characteristics of both proteins and polyphenolics, and thus needs to be deeply investigated.

The main interaction mechanism between the phenolic acids and proteins was the electrostatic attraction. Depending on the temperature, both non-covalent complexations and covalent conjugates can lead to increased antioxidant capacity. Moreover, covalent conjugated forms significantly reduce the digestibility of some food proteins, therefore have an indirect effect on allergen activity. On the other hand, protein-phenolic interactions have direct effect on viral inhibition. These interactions are generally of non-covalent origin and occur between phenolic and non-structural proteins of the viruses. Many phenolics form hydrogen-bonded interactions with those viral proteins and increased number hydrogen bonds result in more stable proteins, which leads to more effective antiviral capacity. In addition to the antioxidant and antiviral activity, dietary phenolic compounds affect both physiology and gene expressions of gut microorganisms as a result of the interaction with microbial proteins. This interaction is not only with commensal microorganism in the gastrointestinal track but also with pathogen microorganisms; so that, they exert antimicrobial activities by inhibiting crucial proteins and microbial growth of such organisms.

Overall, studies on the antioxidant and antimicrobial activities of protein-phenolic conjugates are very limited. Due to the altered structures of both proteins and phenolics, systematic structure-functionality relationships of various conjugates should be investigated together with *in vitro* and *in vivo* studies to fully evaluate the biological activities of protein- phenolic conjugates.

## Author Contributions

HY and BS: conceptualization and methodology. HY, BS, HC, and TO: writing—original draft preparation. HY, BS, HC, and EC: writing—review and editing. EC: supervision. All authors have read and agreed to the published version of the manuscript.

## Conflict of Interest

The authors declare that the research was conducted in the absence of any commercial or financial relationships that could be construed as a potential conflict of interest.

## Publisher's Note

All claims expressed in this article are solely those of the authors and do not necessarily represent those of their affiliated organizations, or those of the publisher, the editors and the reviewers. Any product that may be evaluated in this article, or claim that may be made by its manufacturer, is not guaranteed or endorsed by the publisher.
